# Current Perspectives on Urolithiasis: Pathogenesis, Clinical Management, and Treatment

**DOI:** 10.7759/cureus.101141

**Published:** 2026-01-09

**Authors:** Kamil Wróblewski, Paulina Wróblewska, Sara Szukalska, Marta Karczewska, Karolina Lichwala, Angelika Samborska, Barbara Balajewicz, Lukasz Siwek

**Affiliations:** 1 General Medicine, Poznan University of Medical Sciences, Poznan, POL; 2 General Medicine, Medical University of Silesia, Katowice, POL; 3 Orthopaedics and Traumatology, Dr. Tytus Chałubiński Specialist Hospital in Radom, Radom, POL; 4 General Medicine, Szpital Miejski w Zabrzu, Zabrze, POL

**Keywords:** extracorporeal shock wave lithotripsy (eswl), kidney stones, percutaneous nephrolithotomy (pcnl), renal colic, ureteroscopy (urs), urolithiasis, urology

## Abstract

Urolithiasis is a common and recurrent urological condition with significant clinical and economic implications worldwide. In light of new evidence on evaluating and managing urolithiasis, this review summarizes the current literature and guidelines. The development of urolithiasis is influenced by a complex interplay of dietary, metabolic, environmental, and genetic factors, with calcium-based stones constituting the majority of cases. Patients may present with a wide spectrum of symptoms, ranging from severe renal colic to incidental, asymptomatic findings. Accurate diagnosis requires the appropriate use of laboratory testing and imaging modalities. Non-contrast CT is considered the gold standard for diagnosis, and ultrasonography is recommended as the primary initial tool in many clinical settings. Management strategies depend on stone size, location, symptom severity, and the presence of complications, such as infection or obstruction. Most stones are expelled spontaneously; however, effective treatment options are available, including nonsteroidal anti-inflammatory drugs for pain control, medical expulsive therapy, and minimally invasive procedures such as extracorporeal shock wave lithotripsy, ureteroscopy, and percutaneous nephrolithotomy. These options allow for individualized and highly successful care. Ongoing advances in imaging, medical therapy, and surgical techniques enhance outcomes, reduce recurrence rates, and lessen the overall healthcare burden posed by urolithiasis.

## Introduction and background

Urolithiasis, more commonly referred to as kidney stones, is a prevalent urological condition that can result in pain and complications, such as pyelonephritis or acute renal failure, and often recurs. Appropriate prevention and treatment are of paramount importance, as they contribute to enhancing patients’ quality of life [1-3]. Urolithiasis is a condition in which a stone formed in the kidney moves down the urinary tract to the ureter, bladder, or urethra. The underlying cause of renal calculi is an imbalance between lithogenic mechanisms and stone-forming processes, which gradually leads to their accumulation in the kidney [4]. Patients who have experienced an episode of urolithiasis are at risk of frequent recurrence. Recurrence occurs in half of patients within five years and in as many as 80% within 10 years [5]. The development of urolithiasis is influenced by numerous factors, including geographical location, ethnicity, genetics, and dietary habits. The incidence rate is higher in developed countries and usually ranges from 1% to 20% [6,7]. The predominant composition of urinary stones, accounting for approximately 80% of cases, is calcium oxalate or phosphate. Less common types of stones are composed of uric acid, struvite, or cysteine [8]. Urolithiasis is a significant healthcare problem on a global scale. It is a prevalent disease, and its treatment generates significant financial expenditures. Current estimates indicate that the annual financial burden of urolithiasis management in the United States is anticipated to reach approximately $4.57 billion by the year 2030 [9]. Given the magnitude of the problem, ongoing advancements in diagnostics and treatment methods for urolithiasis are imperative to enhance patient well-being and ensure treatment cost-effectiveness.

## Review

Etiology

A significant proportion of the risk factors for urolithiasis are contingent on the patient’s dietary habits. Factors such as insufficient oral water intake, high animal protein intake, or high oxalate intake (examples of foodstuffs with high amounts of oxalate include beans, soy, beer, cacao, berries, chocolate, some nuts, some teas, coffee, soda, spinach, and potatoes) [8] have been identified as contributing to this condition. The recommended daily fluid intake to prevent kidney stones should be sufficient to produce approximately 2 L of urine. [10]. Ingestion of sodium chloride and other minerals via the oral route is also of significance. As indicated by the extant literature, high salt intake has been demonstrated to be a risk factor for urolithiasis [8]. As demonstrated in research, elevated calcium intake has been shown to induce binding of oxalate within the digestive system, thereby diminishing its absorption into the bloodstream and, consequently, its secretion into urine [11]. Another significant mineral is citrate. In approximately 60% of patients diagnosed with calcium stones, hypocitraturia has been observed. Citrates secreted into the urine form soluble complexes with calcium, thereby preventing stone formation [12,13].

The presence of various medical conditions has been demonstrated to be a significant contributing factor to the development of urolithiasis. These conditions include, but are not limited to, obesity, diabetes mellitus, hypertension, gout, and hyperlipidemia. Metabolic syndrome is a constellation of conditions that includes hypertension, diabetes, obesity, and hyperlipidemia. These conditions are often associated with insulin resistance. Insulin resistance has been demonstrated to result in elevated urinary excretion of calcium, oxalates, and uric acid, which are the fundamental components of kidney stones. Furthermore, obesity has been demonstrated to induce a state of chronic, low-grade inflammation, which, in turn, can lead to a disturbance in solute equilibrium within the urine, ultimately resulting in stone formation. Obese patients have a higher prevalence of low urine pH, which also promotes stone formation. Patients with hyperlipidemia, hypertension, obesity, and type 2 diabetes mellitus frequently consume diets high in salt, sugar, and animal protein, which can serve as an additional risk factor for urolithiasis [14-18].

The prevalence of drug-induced calculi is low, accounting for approximately 2% of all cases. This phenomenon primarily involves protease inhibitors (indinavir and atazanavir) and sulfonamides. These pharmaceuticals, particularly in high doses, have been observed to induce the formation of insoluble compounds that subsequently crystallize within the urine. The stones resulting from protease inhibitors are challenging to visualize on CT scans and are gelatinous in consistency, which complicates diagnosis and renders them resistant to lithotripsy [19].

Furthermore, there is a paucity of research regarding the genetic underpinnings of urolithiasis, with only a few studies having been conducted thus far. A deficiency in renal amino acid and oxalate handling, attributable to metabolic disorders such as cystinuria and primary hyperoxaluria, has been demonstrated to elevate the risk of developing kidney stones [20]. A family history of kidney stones is among the most robust clinical indicators of calculi development [21].

Diagnosis

Patients diagnosed with urolithiasis typically present with severe cramping pain in the lumbar region, vomiting, and fever. However, it is noteworthy that some patients may not present any symptoms [22]. The pain typically manifests and subsequently abates during the acute phase, signifying incomplete obstruction. In cases of severe obstruction, pain is frequently reported during the acute phase. During an episode of renal colic, the pain may radiate to the intestines, groin, bladder, or genitalia due to dual innervation of the genitourinary, gastrointestinal, and somatic systems [23]. As indicated by the extant literature, vomiting and nausea are observed in approximately 50% of patients diagnosed with urolithiasis [24].

As the stone traverses the ureter, it has the potential to induce hematuria, dysuria, or sudden urgency. Patients afflicted with renal calculi typically do not present any symptoms. The presence of blood in the urine, known as hematuria, is typically microscopic in nature, accounting for approximately 90% of cases. However, under certain circumstances, it can manifest as macroscopic, which is characterized by the presence of blood cells visible to the naked eye. While hematuria is a significant symptom, its absence does not allow for the exclusion of urolithiasis [23]. The sensitivity of hematuria is highest on the first day of symptoms (approximately 95%), but decreases after three to four days to 65% [25]. Some patients experience elevated blood pressure and tachycardia associated with severe pain [23]. The presence of fever and chills is typically indicative of infected calculi. The presence of urolithiasis in conjunction with urinary tract infection has been demonstrated to result in a considerable escalation in the probability of developing urosepsis. This condition necessitates the expeditious initiation of consultation for admission and emergent urologic intervention [23,26]. The presence of costovertebral angle tenderness is frequently identified during physical examinations. An abdominal examination typically reveals no abnormalities, although there may be signs of guarding and tenderness [27].

Imaging is the prevailing modality for diagnosing urolithiasis; however, laboratory tests can play a pivotal role in evaluating the patient’s condition and determining the necessity for emergent surgical intervention. Some patients exhibit reduced creatinine clearance, though rarely to a degree significant enough to qualify as acute renal injury. The most common cause of acute renal injury in urolithiasis is dehydration from vomiting [28,29]. Females of reproductive age must undergo pregnancy testing [28]. A urinalysis may reveal the presence of hematuria or elevated white blood cell (WBC) counts. The presence of WBCs in the urine does not necessarily indicate a urinary tract infection. The presence of pyuria can be indicative of underlying urethral inflammation. However, an elevated concentration of WBCs in urine is associated with an increased risk of urinary tract infection [23]. The presence of 10-20 WBCs per high-powered field (hpf) has been associated with a 9.1% probability of urinary tract infection, while for more than 50 WBCs/hpf, the probability increases to 60% [30].

The foundation for diagnosing urolithiasis is imaging, which may include ultrasound, X-rays, CT, or MRI. An X-ray of the kidney, ureter, and bladder can be useful for assessing calcium-containing stones, which are radiopaque. However, the presence of radiolucent deposits, typically composed of uric acid or cystine, poses a significant challenge in assessment. Furthermore, the sensitivity and specificity of the test have been documented to range from 59% to 71%. Moreover, X-rays are not employed to evaluate hydronephrosis and the precise location of the stone, which is imperative in confirming the diagnosis of renal colic [31]. Due to these limitations, X-rays are rarely used in urolithiasis.

Non-contrast CT (NCCT) is widely regarded as the gold-standard imaging technique for the diagnosis of urolithiasis, with reported sensitivity and specificity rates ranging from 95% to 100% [31]. NCCT facilitates the evaluation of the stone’s location, size, and density, in addition to its type and the prediction of its susceptibility to lithotripsy. The technology has been demonstrated to facilitate the identification of hydronephrosis and enable the exploration of other potential etiologies contributing to patient symptoms [23]. The most significant disadvantage associated with this procedure is that it exposes the patient to radiation, which is not recommended for certain groups of patients, such as pregnant women. Furthermore, NCCT lacks the capacity to visualize stones that are formed as a secondary complication of protease inhibitor use [19]. The utilization of low-dose NCCT has been demonstrated to result in a reduction of radiation exposure. However, this approach exhibits reduced sensitivity in detecting small stones measuring less than 3 mm (86%) and in individuals with a body mass index greater than 30 kg/m² [32,33]. In cases where an alternative etiology is suspected, contrast CT may be considered. The modality exhibits comparable sensitivity in detecting stones and has the capacity to more effectively detect urinary tract strictures. However, it should be noted that the procedure is time-consuming and carries an increased risk of kidney damage [34,35].

In accordance with the European Association of Urology (EAU) Guidelines on Urolithiasis from 2025, ultrasound is to be utilized as the primary imaging diagnostic modality. However, additional imaging procedures and other emergency interventions must not be delayed [36]. Ultrasound is a radiological imaging modality that poses no inherent risk of radiation exposure. It is a rapid and cost-effective procedure. While ultrasound boasts a high degree of specificity (97.5%), its sensitivity (57%) for diagnosing ureteric stones is comparatively low. Consequently, it is not recommended to rely solely on ultrasound as a diagnostic imaging modality [37]. Ultrasonography has been demonstrated to indirectly reveal urolithiasis by detecting hydronephrosis, which is present in 89% of patients [38]. The absence of hydronephrosis in urolithiasis may be attributable to dehydration and frequently occurs subsequent to intravenous fluid therapy [39]. The absence of hydronephrosis has been shown to reduce the likelihood of urological intervention [40], while a higher degree of hydronephrosis has been associated with a larger stone [38].

MRI does not expose patients to radiation and can be a useful imaging modality for pregnant women [23,33]. MRI has been proposed as a diagnostic tool with a reported 93% accuracy rate [41]. MRI’s prolonged duration, substantial expense, and constrained accessibility have collectively diminished its role in the diagnosis of renal calculi, relative to alternative imaging modalities [33].

Treatment and management

The therapeutic approach for urolithiasis is contingent upon the patient’s symptoms and may encompass both medical and surgical interventions. Patients afflicted with renal colic frequently exhibit symptoms of acute pain, which should be addressed with utmost urgency. The underlying etiology of renal colic pain is attributed to the obstruction of urine flow and subsequent tension in the walls of the ureter. This results in an elevated release of prostaglandins, which, in turn, leads to an increase in pain and inflammation [42,43]. Nonsteroidal anti-inflammatory drugs (NSAIDs) have been shown to possess analgesic properties and to impede prostaglandin synthesis, thereby enhancing their efficacy in comparison to opioids in the management of renal colic [44]. The treatment of nausea and vomiting can be addressed through the administration of antiemetic medications such as ondansetron or metoclopramide. A comparative analysis of the efficacy of these medications has been conducted, with ondansetron demonstrating superiority over metoclopramide in terms of effectiveness [45]. Alpha-adrenergic antagonists, such as doxazosin and tamsulosin, have been shown to reduce the duration of the process and enhance the stone passage rate. Furthermore, these interventions have been demonstrated to reduce analgesic requirements and minimize the risk of hospitalization. The aforementioned effects do not apply to stones measuring less than 5 mm [46-48]. Intravenous fluids have been demonstrated to increase the volume of urine excreted; however, they do not facilitate stone passage. These substances should be reserved for the treatment of dehydration [49]. Research indicates that 86% of renal calculi are spontaneously expelled. The size of the stone directly correlates with the probability of spontaneous expulsion, with larger stones exhibiting a lower likelihood of such events [50].

The decision to perform urological intervention in urolithiasis is contingent upon a multitude of factors, including stone size and location, patient symptoms, urinary tract obstruction, and the presence of infection. The most prevalent methodologies employed in the management of urolithiasis encompass extracorporeal shock wave lithotripsy (ESWL), ureteroscopy (URS), and percutaneous nephrolithotomy (PCNL). Acute renal obstruction necessitates prompt decompression to avert irreversible renal injury. Decompression can be achieved through the use of a ureteral catheter or a percutaneous nephrostomy procedure [51].

ESWL is a procedure that utilizes shock waves to fracture kidney stones into fragments that are then eliminated through urination. This method has been demonstrated to be applicable to renal stones, as well as ureteric stones, with a particular emphasis on the proximal ureteric segments. The effectiveness of the procedure has been documented to reach up to 75%, though it is acknowledged that certain factors, including obesity, have the potential to markedly reduce its efficacy. It is important to note that cystine stones may not respond to treatment. ESWL is a noninvasive treatment that can be performed on an outpatient basis. This approach has been demonstrated to be significantly more cost-effective [51,52].

URS is a minimally invasive technique that utilizes a flexible or rigid ureteroscope, which is inserted through the urethra to provide direct visualization of the stone and facilitate laser lithotripsy-induced fragmentation. URS has been demonstrated to be a highly effective treatment for ureteral and kidney stones, particularly those measuring less than 1 cm in size, with a success rate exceeding 90%. The objective of this procedure is the complete removal of the stone. The current recommendation is against the routine pretreatment of the ureters before URS. The EAU does not recommend routine stenting before and after URS due to the lack of significant improvement in patient outcomes and poor cost-effectiveness. In patients deemed to be at high risk of complications, the utilization of stenting may be contemplated as a strategy to avert the necessity for repeated interventions in emergencies. However, the administration of alpha-blockers before the procedure, specifically one week prior, has been observed to potentially enhance the stone-free rate. As is the case with many endoscopic procedures, antibiotic prophylaxis is recommended to reduce the risk of developing a urinary tract infection. The holmium:yttrium-aluminum-garnet laser is regarded as the gold standard due to its ability to enhance efficacy and safety, in addition to its capacity to effectively address all types of calculi. Furthermore, the thulium fiber laser has been observed to demonstrate a reduced propensity for stone migration into the kidney in certain cases. URS is considered to be a low-risk procedure; nevertheless, as with any surgical intervention, the potential for complications exists. The complication rate following URS has been reported to range from 4% to 25%, with the majority of adverse events being mild and not necessitating additional intervention. The most severe cases include postoperative strictures, ureteral injury, and perforation. The most severe complication is ureteral avulsion, with a rate ranging from 0.06% to 0.45%. URS is also a valuable alternative for patients who cannot tolerate anticoagulant or antiplatelet drugs [36,53].

The procedure of PCNL entails acquiring percutaneous access to the kidney, the subsequent dilation of the tract, and the securing of the tract with a sheath. The insertion of a nephroscope through the sheath facilitates visualization, fragmentation, and elimination of renal calculi. PCNL is considered the primary treatment for stones measuring 2 cm or greater. It is regarded as the gold standard for the treatment of staghorn calculi, with a documented success rate of 92%. It is recommended for patients who have failed ESWL or URS, or who have a history of chronic kidney disease. It is noteworthy to mention a mini-PCNL, which has a smaller sheath diameter. Mini-PCNL has been shown to have a comparable stone-free rate and has been associated with minimal blood loss and a reduced length of hospital stay. Despite its high effectiveness, PCNL carries a higher risk of complications than minimally invasive methods. Postoperative bleeding is a frequent occurrence, with approximately 7% of patients requiring postoperative blood transfusions. Contraindications to PCNL include anticoagulant therapy, current pregnancy, active urinary tract infection, and suspected malignant tumors in the kidney or in the access tract [36,54].

## Conclusions

Urolithiasis is a prevalent and clinically significant condition with substantial implications for patient morbidity and global healthcare systems. The predominant presentation of this condition involves severe flank pain, hematuria, nausea, and vomiting. The etiology of the condition is multifactorial, encompassing dietary habits, metabolic disorders, genetic predisposition, and certain medications. This multifactorial etiology underscores the necessity of comprehensive risk assessment and the development of individualized prevention strategies. The accuracy and timeliness of diagnosis are contingent upon an appropriate combination of clinical evaluation, laboratory testing, and imaging modalities. Non-contrast CT and ultrasonography serve as cornerstone tools in this regard. A customized management approach is imperative, taking into account stone characteristics, symptom severity, and the presence of complications, including infection or obstruction. The majority of stones pass spontaneously, and those that require surgical intervention can be treated using minimally invasive methods such as ESWL, URS, and PCNL, which have improved safety profiles. Continued refinement of diagnostic algorithms, optimization of medical expulsive therapies, and innovation in surgical techniques remain essential to reducing recurrence, improving patient quality of life, and minimizing the economic burden of this widespread disease.
